# Expression of human papillomavirus 6b L1 protein in silkworm larvae and enhanced green fluorescent protein displaying on its virus-like particles

**DOI:** 10.1186/2193-1801-1-29

**Published:** 2012-10-04

**Authors:** Muthukutty Palaniyandi, Tatsuya Kato, Enoch Y Park

**Affiliations:** 1 Laboratory of Biotechnology, Integrated Bioscience Section, Graduate School of Science and Technology, Shizuoka University, 836 Ohya, Suruga-ku, Shizuoka, 422-8529 Japan; 2 Laboratory of Biotechnology, Department of Applied Biological Sciences, Faculty of Agriculture, Shizuoka University, 836 Ohya, Suruga-ku, Shizuoka, 422-8529 Japan

**Keywords:** Virus-like particles, Human papillomavirus silkworm expression system, BmNPV bacmid

## Abstract

Human papillomavirus (HPV) 6b L1 capsid protein was expressed using the *Bombyx mori* nucleopolyhedrovirus (BmNPV) bacmid expression system in silkworm larvae. Two constructs, full-length L1 (500 a.a) and C-terminal-deleted short L1 (479 a.a), and three PCR-manipulated antigenic loops at amino acids 55–56, 174–175, and 348–349 regions were incorporated with whole enhanced green fluorescent protein (EGFP). Expressed in full, short L1 proteins and variants were purified in heparin affinity column chromatography and confirmed by SDS-PAGE and western blot. The presence of self-assembled virus-like particles (VLPs) and EGFP incorporation on the surface of VLPs were confirmed by the observation of transmission electron and immunoelectron microscopies, respectively. HPV 6b L1 major capsid protein was successfully expressed in silkworm, and effective manipulation on the antigenic regions showed the path to versatile vaccine development based on HPV L1-VLPs.

## Background

Virus-like particles (VLPs) are empty virus particles, which lack virus-derived genome DNA or RNA, and composed of virus-capsid or matrix proteins. A non-enveloped virus, VLPs are mainly composed of virus-capsid proteins, which can be self-assembled both in vitro and in vivo. VLPs have been utilized as a vaccine because of its high immunogenicity and capability of inducing cellular and humoral immune responses ([Grgacic and Anderson 2006]). Alternatively, VLPs can be used as functional nanoparticles for drug delivery system, cell and tissue imaging by modification of the surface of VLPs and incorporation of functional materials into VLPs (Georgens et al. [2005]).

To modify the surface of VLPs, chemical and genetic modifications have been adopted (Ma et al. [2012]; [Pokorski and Steinmetz 2011]). An enhanced green fluorescent protein (EGFP) variant and outer surface protein C (OspC) from *Borrelia burgdorferi* were inserted into the immunodominant c/e1 epitope region of hepatitis B virus capsid protein without the disruption of VLP’s shape (Kratz et al. [1999]; Nassal et al. [2008]). In general, it is difficult to display a whole full-length protein on the surface of VLPs by genetic modification.

In this study, human papillomavirus (HPV) 6b L1 protein was expressed in silkworm larvae, and HPV 6b L1-VLPs were purified from the fat body of silkworms. HPV is a non-enveloped virus that has approximately 50–60 nm in diameter and ~8 kbp of double-stranded circular DNA genome. HPV L1 protein can be self-assembled as a VLP when this protein is expressed in various expression systems (Trus et al. [1997]). To display this whole protein on the surface of HPV 6b L1-VLPs, EGFP, as a model of a whole full-length protein, was inserted into BC, EF, and HI loop domains (Bishop et al. [2007]; Chen et al. [2000]) of HPV 6b L1 protein. EGFP-incorporated HPV-VLPs were analyzed and discussed targeting for using alternative vaccine candidate.

## Results and discussion

### Expression of HPV 6b L1 protein and its short variant in silkworm larvae

**Figure 1:**
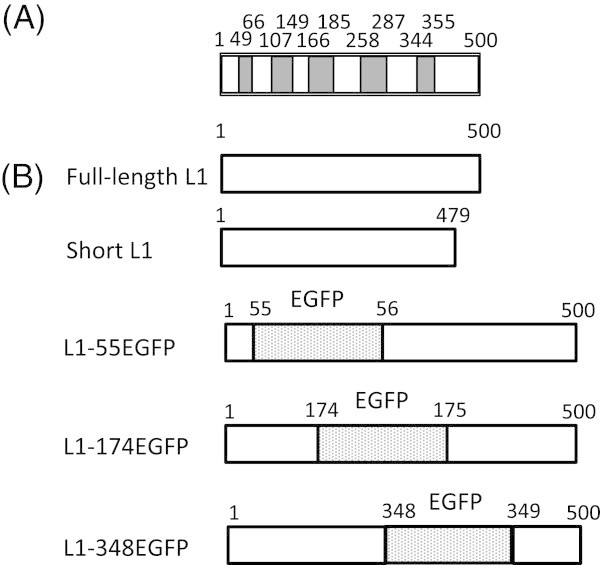
**Genetic constructs of HPV 6b L1 (A) and its variants (B) proteins.** Numbers in the grey boxes of (**A**) and (**B**) indicate loop regions and amino acid residues of L1 protein.

**Figure 2:**
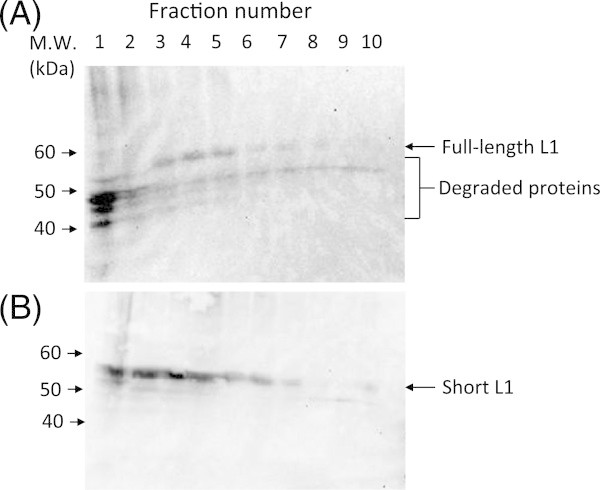
**Sucrose density-gradient centrifugation analysis of HPV 6b L1 full- (A) and short-proteins (B) expressed in the fat body of silkworm larvae.** Fat body was suspended with homogenate buffer and sonicated. The homogenate was centrifuged to remove insoluble materials and its supernatant was analyzed by sucrose density-gradient centrifugation.

HPV 6b L1 protein has a basic domain at its C-terminus, which can bind to genomic DNA and pack into virus particles (Li et al. [1997]; Touze et al. [2000]). Full-length L1 and short proteins lacking its basic domain at its C-terminus (Figure 1B) were expressed in silkworm larvae. Both the L1 protein and its variant were expressed in the fat body of silkworm larvae, but not in hemolymph (data not shown). In sucrose density gradient centrifugation analysis, many bands that derived from L1 full-length protein were detected mainly in fraction 1, but these bands have lower molecular weight than that of full-length L1 (Figure 2A). These low-molecular weight L1 proteins may come from proteolytic degradation by some proteases in silkworms. Full-length L1 protein was detected mainly in between fractions 3 and 5. On the other hand, short L1 protein was observed mainly in between fractions 1 and 4 (Figure 2B). Full-length L1 protein was observed in a slightly higher sucrose density fraction compared to short L1 protein, suggesting that full-length L1 protein may form large particles.

**Figure 3:**
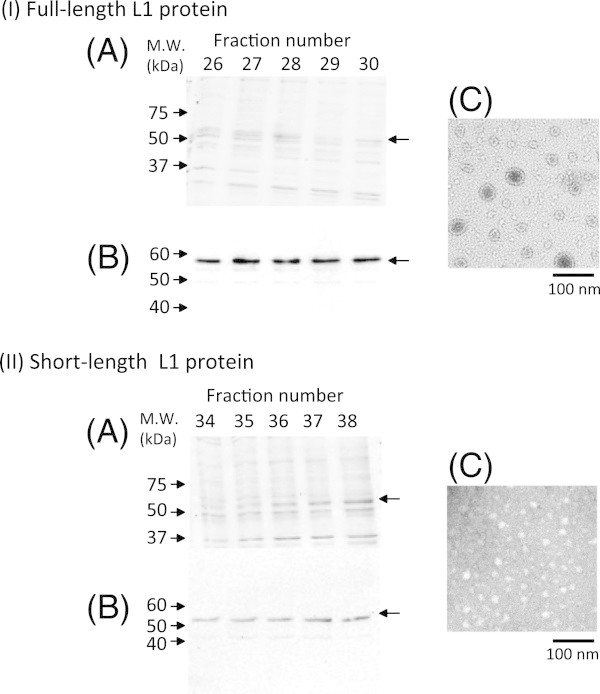
**Analysis of partial purified full-length (I) and short L1 (II) proteins.** (**A**) SDS-PAGE analysis of heparin affinity column chromatography with CBB staining. (**B**) Western blot analysis of heparin affinity column chromatography. (**C**) Electron micrographs of partially purified proteins.

After the partial purification of full-length and short L1 proteins from the fat body of silkworm larvae by heparin affinity column chromatography, both purified full-length and short L1 proteins were analyzed by SDS-PAGE, western blot, and electron microscope. Minor low-molecular weight bands were detected by SDS-PAGE (Figure 3I-A and 3II-A), and their molecular weights were also confirmed by western blot (Figure 3I-B and 3II-B). Full-length L1 proteins formed heterogenous VLPs, which have 10–50 nm in diameter (Figure 3I-C). HPV-VLPs have a diameter of 55 nm, suggesting that large VLPs have a normal size of HPV-VLPs in a T=7 icosahedral lattice, which consists of 72 pentamers, and small VLPs are T=1 particles, which consist of 12 pentamers or capsomeres. On the other hand, short L1 protein formed only small particles (Figure 3II-C). This result suggests that C-terminal basic domain in L1 protein may be required to form T=7 particles. Touzé et al. previously reported that Δ31 L1 protein from HPV 16, where 31 amino acids at its C-terminus were deleted, were still able to form T=7 particles, but deletion of 103 amino acids at its C-terminus caused the disruption of L1-VLP formation (Touze et al. [2000]). Moreover, the deletion of 22 amino acids in HPV 16 L1 protein did not have any effect on the assembly of T=7 VLPs. Most of the C-terminal-deleted L1 variant formed T=7 VLPs (Varsani et al. [2006]). Bian et al. reported that HPV 16 L1 ΔC34 (deleted 34 amino acids at its C-terminus) fused with an N-terminal domain (1–70 amino acids) of HPV E7 formed 11–12 nm of capsomeres, but not as large as VLPs (Bian et al. [2008]). These previous reports were obtained from the *Escherichia coli* expression system, but in this study, bacmid-silkworm expression system was used. These results suggest that protein expression system may have some influences on the properties of expressed L1 proteins.

### Expression of L1 protein inserted with EGFP at its loop domain

**Figure 4:**
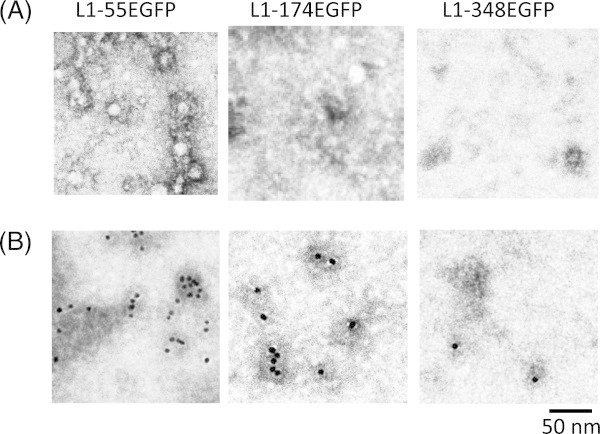
**Electron micrographs (A) and immunoelectron micrographs (B) of each partially purified L1-EGFP fusion proteins.** Detailed methods are described under Materials and methods.

To date, an antigen display system, found on the surface of VLPs targeting for efficient vaccines to infectious diseases, has been established using chemical conjugation, gene manipulation for the identification of immunogenic domains, conformation, and neutralizing epitopes of capsid proteins. Crystal structures of HPV 11, 16, 18, and 35 L1 proteins have been already solved, and L1 proteins have 5 loop regions (BC, DE, EF, FG, and HI loops) (Bishop et al. [2007]). Chimeric HPV 16 L1 proteins were constructed by the introduction of six amino acids coding hepatitis B core (HBc) antigen epitope (DPASRE) into 5 loop regions in HPV 16 L1 protein (Sadeyen et al. [2003]). These chimera L1 protein-formed T=7 particles and induced anti-HBc antibodies indicate that HBc epitope can be displayed on HPV VLP’s surface by its insertion into the loop region of L1 protein. Moreover, several heterogenous epitopes were also displayed on its surface by chemical conjugation and genetic manipulation (Murata et al. [2009]; Pejawar-Gaddy et al. [2010]; Schellenbacher et al. [2009]). In this study, whole protein was inserted into BC, EF, and HI loops of HPV 6b L1 protein by genetic manipulation. The alignment search was performed between the amino acid sequences of HPV 16 L1 and 6b L1 proteins, and putative each loop region in HPV 6b was described in Figure 1A. Each fusion protein (L1-55-EGFP, L1-174-EGFP, and L1-348-EGFP) was constructed as Figure 1B, and expressed in silkworm larvae. Three fusion proteins were detected by anti-L1 antibody and anti-EGFP antibody in the fat body at the estimated molecular weight, but most of the expressed protein was degraded (data not shown). These three fusion proteins were partially purified by heparin affinity column chromatography using almost the same protocol as the purification of full-length and short HPV L1 proteins, and were analyzed by electron microscope (Figure 4A). Approximately 10–20 nm of particles, capsomeres, and amorphous aggregates were observed in all samples, but VLP particles were not. By immunoelectron microscopy using an anti-EGFP antibody as the primary antibody, gold particles were observed on the capsomeres and aggregates in all samples (Figure 4B). EGFP fluorescence of partially purified L1-EGFP fusion protein was observed, but all L1-EGFP fusion protein did not have any fluorescence. These results indicate that L1-EGFP fusion proteins form capsomeres, and EGFP can be displayed on the surface of L1 capsomere without native EGFP conformation. In a previous report, 95 amino acids (HPV 16 L2 13–107 amino acids) were genetically inserted into the DE loop in bovine papillomavirus (BPV), and this L1 fusion protein did not form unequivocal VLPs but formed capsomeres and its aggregates. However, this fusion protein induced a slight but significant antibody response to HPV L2 protein when this L1-L2 fusion protein was immunized to rabbits with a native form (Schellenbacher et al. [2009]). These results suggest that it is possible that the insertion of long polypeptide (100 a.a.~) into the loop domain in HPV L1 protein disturbs VLP formation, but L1 protein inserted with a long polypeptide forms capsomeres and its L1-long polypeptide fusion protein capsomeres are prone to aggregate. In this study, three fusion proteins formed aggregates.

## Conclusions

Three L1-EGFP fusion proteins did not have green fluorescence, but EGFP was displayed on the surface of its capsomeres. To date, a genetic whole protein display system on the VLP has not been established, except for hepatitis B virus nucleocapsid protein VLP (Kratz et al. [1999]; Nassal et al. [2008]). These results suggest that this whole protein display system using HPV L1 protein can be applied to develop new vaccines against infectious diseases, and HPV L1 protein can serve as a potential vaccine template.

## Methods

### Construction of recombinant BmNPV bacmids

HPV 6b L1 gene-containing plasmid vector was purchased from the American Type Culture Collection (ATCC). HPV 6b L1 full-length gene (a.a. 1–500, Figure 1A) was amplified by PCR using primers HPV 6b L1-F and HPV 6b L1-R (Table 1). HPV 6b L1 short gene (a.a. 1–479) was amplified by PCR using primers HPV 6b L1-F and HPV 6b L1 short-R (Table 1). Each amplified fragment was purified and inserted into pENTR/D/TOPO (Invitrogen, Carlsberg, USA). The construction of recombinant BmNPV CP^-^ bacmid containing full-length L1 gene or short L1 gene was performed according to a previous report (Hiyoshi et al. [2007]).

**Table 1:** **Primers for amplification of the HPV L1 gene and chimeric variants**

Primers	5’ – 3’
HPV 6b L1-F	CACCATGTGGCGGCCTCGCGACAGC
HPV 6b L1-R	TTACCTTTTAGTTTTGGCGCG
HPV 6b L1 short-R	TTAAACACCTGTACGAATAGAGG
Eco-HPV 6b L1-F	CGGAATTCATGTGGCGGCCTCGCGACAGC
HPV 6b L1-55R-LINKER	TGTACCACCACCACCGCTACCACCACCACCGTTAGCCCGTTTTATGGA
HPV 6b L1 LINKER-56F	GGAGGAGGAGGAAGCGGAGGAGGAGGAAAAACTGTTGTGCCAAAGGTG
Kpn-HPV 6b L1-R	GGGGTACCTTACCTTTTAGTTTTGGCGCG
HPV 6b L1-174R-LINKER	TGTACCACCACCACCGCTACCACCACCACCAGGTGTATTAGTACACTG
HPV 6b L1 LINKER-175F	GGAGGAGGAGGAAGCGGAGGAGGAGGAGTACAGGCTGGTGACTGCCCG
HPV 6b L1-348R-LINKER	TGTACCACCACCACCGCTACCACCACCACCGGAAGATGTAGTTACGGA
HPV 6b L1 LINKER-349F	GGAGGAGGAGGAAGCGGAGGAGGAGGAACATACACCAATTCTGATTAT
LINKER-EGFP-F	GGTGGTGGTGGTAGCGGTGGTGGTGGTACAATGGTGAGCAAGGGCGAG
EGFP-R-LINKER	TCCTCCTCCTCCGCTTCCTCCTCCTCCCTTGTACAGCTCGTCCATGCC

Each L1-EGFP fusion gene was obtained by two-step PCR. To obtain L1-EGFP55, two truncated L1 genes coding a.a.1–55 and 56–500 were amplified by PCR using primers Eco-HPV 6b L1-F and HPV 6b L1-55R-LINKER and HPV 6b L1 LINKER-56F and Kpn-HPV 6b L1-R (Table 1), respectively. In addition, an EGFP gene was amplified by PCR using primers LINKER-EGFP-F and EGFP-R-LINKER (Table 1). A second PCR was performed using the amplified EGFP gene as a template and the two amplified truncated L1 gene as primers to obtain L1-EGFP55 fusion gene. The EGFP gene was inserted between 55 and 56 amino acids in an L1-coding gene. Moreover, linker-region coding sequences (GGGGSGGGGS) were also added between L1 and EGFP genes. By this two-step PCR, L1-EGFP174 and L1-EGFP348 were obtained by PCR using primers (Table 1). Each amplified fusion gene was inserted at *Eco*RI-*Kpn*I site in pFastBac1. The recombinant BmNPV CP^-^ bacmid containing each L1-EGFP fusion gene was constructed according to the protocol described above.

### Expression of HPV 6b L1 full- and short-protein and variants in silkworm

A recombinant BmNPV CP^-^ bacmid was prepared by alkaline extraction described in the Bac-to-Bac manual (Invitrogen). Five micrograms of extracted BmNPV CP^-^ bacmid DNA, together with a helper plasmid, was mixed with 1/10 volume of DMRIE-C reagent (Invitrogen) and incubated at room temperature for an hour. This mixture was injected into silkworm larvae. The DNA-injected silkworm larvae were reared using Silkmate 2S (NOSAN Co. Yokohama, Japan) as a diet for 6–7 days and followed by the collection of fat body from silkworm larvae. Collected fat body was stored at −80°C before use.

### Partial purification of HPV VLPs

The fat body was suspended in a homogenate buffer (50 mM Tris–HCl buffer [pH 7.5] containing 150 mM NaCl) and sonicated to extract expressed proteins. The homogenate was centrifuged at 30000 × g for 15 min. A supernatant was applied to the HiTrap heparin affinity column chromatography (GE Healthcare, Pittsburgh, PA, USA). After loading the sample, the column was washed with 20-column volumes of homogenate buffer, followed by an elution by NaCl concentration gradient to 2 M. Fractions containing L1 protein were collected and dialyzed against homogenate buffer overnight. The dialyzed sample was applied to Mono S 5/50GL column chromatography (GE Healthcare). The column was washed with 10-column volumes of homogenate buffer, and proteins were eluted by NaCl concentration gradient to 1 M.

### Sodium dodecyl sulfate-polyacrylamide gel electrophoresis (SDS-PAGE) and western blot analysis

The samples were subjected to SDS-PAGE on a 10 or 12% polyacrylamide gel with the Mini-protean II system (Bio-Rad, Hercules, CA, USA). The total number of proteins on the SDS-PAGE gel was detected with Coomassie Brilliant Blue (CBB) R-250. In the case of western blot, the proteins found in the gels were blotted onto a polyvinylidene fluoride (PVDF) membrane using the Mini Trans-Blot Electrophoretic Transfer Cell (Bio-Rad). After being blocked in 5% skim milk in a Tris-buffered saline containing 0.1% Tween 20 (TBST), the membrane was incubated in a 1:10000 diluted mouse anti-HPV 16 L1 antibody (Novus Biologicals Inc. Littleton, CO, USA) for an hour. This antibody can also recognize HPV 6b L1 protein. The membrane was washed with TBST and then incubated in a 1:20000 diluted either anti-mouse or anti-rabbit labeled with horseradish peroxidase (HRP) (GE Healthcare) for an hour. Detection was performed using ECL Plus Western Blotting Reagent (GE Healthcare). Specific bands were detected using a Fluor-S/MAX Multi-Imager (Bio-Rad). Protein band intensity was analyzed by Quantity One software (Bio-Rad).

### Sucrose density gradient centrifugation analysis

The homogenate of the fat body was laid on a 25–60% sucrose density gradient. This suspension was centrifuged at 96000 × g for 3 h at 4°C. Each 0.5 ml of the fraction was collected at the top of the tube. Each fraction was analyzed by SDS-PAGE and western blot.

### Transmission electron and immunoelectron microscopic analyses

Purified VLPs were immobilized on the grid and blocked with 4% BSA in PBS (pH 7.4). This grid was soaked in either mouse anti-HPV 16 L1 antibody diluted by 30-fold with 1% BSA in PBS for 2 h. After washing with PBS, the grid was soaked in either 10 nm gold-conjugated goat polyclonal anti-mouse IgG+IgM (H+L) (British BioCell International, Cardiff, UK) diluted by 25-fold with 1% BSA in PBS for 1 h. After washing with PBS, the grid was stained with 2% phosphotungstic acid. VLPs were observed by JEM-2100F (JEOL Ltd., Japan) at 200 kV.
